# MiRNAs Regulating Insulin Sensitivity Are Dysregulated in Polycystic Ovary Syndrome (PCOS) Ovaries and Are Associated With Markers of Inflammation and Insulin Sensitivity

**DOI:** 10.3389/fendo.2019.00879

**Published:** 2019-12-13

**Authors:** Francesca Cirillo, Cecilia Catellani, Pietro Lazzeroni, Chiara Sartori, Alessia Nicoli, Sergio Amarri, Giovanni Battista La Sala, Maria Elisabeth Street

**Affiliations:** ^1^Division of Pediatric Endocrinology and Diabetology, Department of Mother and Child, Azienda USL–IRCCS di Reggio Emilia, Reggio Emilia, Italy; ^2^Center of Reproductive Medicine and Surgery, Department Mother and Child, Azienda USL–IRCCS di Reggio Emilia, Reggio Emilia, Italy

**Keywords:** miRNA, PCOS, inflammation, HMGB1, insulin resistance, estradiol

## Abstract

**Objective:** MicroRNAs (miRNAs) are gene expression regulators. Altered miRNA levels are associated with diabetes, insulin resistance, and inflammation. Insulin resistance and inflammation are both features of Polycystic ovary syndrome (PCOS). The aim of this study was first to assess differences in selected miRNAs (miR-146a, miR-155, miR-320, miR-370, miR-486), involved in insulin sensitivity regulation and inflammation, in women with or without PCOS. Second, to investigate relationships among these miRNAs, insulin, High mobility group box 1 (HMGB1), and IL-6 in follicular fluid (FF), serum 17-beta estradiol (E2), and the number of dominant follicles.

**Methods:** Thirty PCOS and thirty-six non-PCOS women undergoing *in vitro* fertilization were enrolled. RNA from granulosa cells (GC) and FF was extracted and the specific miRNAs were evaluated using qRT-PCR. HMGB1, insulin, and IL-6 in FF, and serum E2 were assayed using specific kits.

**Results:** MiR-146a, miR-155, miR-486 were upregulated and miR-320 and miR-370 were downregulated in GC from the PCOS patients. In FF, miR-146a, miR-155, and miR-486 showed lower levels in PCOS, whereas miR-320 and miR-370 showed an opposite trend but no significant changes were observed. These miRNAs showed relationships with Body Mass Index (BMI), age, E2, number of dominant follicles, insulin, and HMGB1.

**Conclusion:** In conclusion, the miRNAs analyzed showed changes in PCOS ovaries and had relationships with indices of inflammation and insulin sensitivity within the ovary, providing evidence for new regulatory mechanisms.

## Introduction

Polycystic ovary syndrome (PCOS) is a common multifactorial and heterogeneous endocrine disorder affecting women of reproductive age (1). Although its etiology remains unclear, environmental, genetic and epi-genetic factors contribute to this disorder.

PCOS is characterized by hyperandrogenism, ovulatory dysfunction, and polycystic ovarian morphology (1). Although insulin resistance (IR) is not considered as a diagnostic criterion, it underpins the disease in 50–70% of women independent of Body Mass Index (BMI) (2). Furthermore, insulin sensitizing treatments, as metformin, have often proved to be effective (3) and in recent years inositol derivatives (4, 5) and alpha lipoic acid have shown positive effects besides being safe (6, 7). The mechanisms related with the regulation of insulin sensitivity at the ovarian level are not fully elucidated yet. Definitely an important role has been described relative to Peroxisome proliferator-activated receptors (PPARs) (8–10).

In recent years, it has been reported that PCOS is associated with systemic low-grade inflammation (LGI) (11–14). Recent evidence reports that intrafollicular inflammatory mediators are enhanced in periovulatory follicles of PCOS women (15), and chronic LGI might be a precursor of ovarian dysfunction in PCOS. In addition, High mobility group box 1 (HMGB1), a small protein with cytokine activity (16), is increased in follicular fluid (FF) from ovaries of PCOS women (17) in relationship with decreased Cystic fibrosis transmembrane conductance regulator (*CFTR*) expression in granulosa cells (17), as previously described in Cystic Fibrosis (CF) (18, 19). Serum HMGB1 levels have been reported to be higher in PCOS women with IR (17, 20).

MicroRNAs (miRNAs) represent a recent chapter of epigenetics and have become useful for the comprehension of multiple diseases offering new insights into the molecular mechanisms. They consist in endogenous small single stranded non-coding RNAs, ~22 nucleotides long, which act as post transcriptional regulators. A single miRNA can act on several hundreds of target mRNAs and each mRNA can be the target of many miRNAs (21, 22). Upon biosynthesis, they can be released into the extracellular space and appear remarkably stable in various bodily fluid such as FF, which reflects the secretory and metabolic activities of oocytes and follicle niche (23). Therefore, altered levels of miRNAs could affect/reflect ovarian insulin sensitivity, hormone synthesis, and inflammation. However, few studies, and with contradictory results, have been conducted in humans relative to miRNAs in the different ovarian compartments (24).

We selected miR-146a, miR-155, miR-320, miR-370, and miR-486 involved both in insulin sensitivity and in chronic inflammation and regulated in serum of CF patients at onset of CF related diabetes (25).

MiR-146a is increased *in vitro* under inflammatory stimulation (26), and is involved in insulin resistance in type 2 diabetic patients (27). MiR-155 has a well-documented role in autoimmune and other chronic inflammatory diseases (28–31). Furthermore, miR-155 has been demonstrated to regulate insulin sensitivity *in vitro* (32) and *in vivo* in mice (32, 33). MiR-320 is currently considered as a potential target for type 2 diabetes mellitus therapy (34, 35) since it regulates the expression of phosphoinositide-3-kinase (*PI3K*), a downstream mediator of insulin signaling. Moreover, miR-320 regulates the expression of *NOD2* a cytosolic receptor involved in the proinflammatory cascades in chronic inflammatory bowel diseases (36). MiR-370 is a modulator of *IRS1* expression, a scaffold protein involved in the insulin pathway (37, 38). MiR-486 directly targets mediators of insulin-like growth factor (*IGF*) signaling including *IGF-I*, IGF-I receptor (*IGF1R*), and PI3K regulatory subunit 1 (alpha) (*PIK3R1*) and is reduced in plasma of diabetic patients (39, 40).

The aim of this study was first to assess differences in the selected miRNAs, in women with or without PCOS undergoing *in vitro* fertilization (IVF); second to investigate relationships among these miRNAs, HMGB1, insulin, IL6 in FF, and 17-beta estradiol (E2) in serum.

## Subjects and Methods

### Patients

In the present study we enrolled 30 female patients with PCOS and 36 regularly cycling women as controls (CTRL) matched for age and BMI as previously described (17). All subjects were enrolled at the time of oocyte retrieval at IVF center of our Institution. Details concerning the hormonal stimulation protocol are specified below. PCOS patients (CA: 34.43 ± 0.84 yr; BMI: 25.92 ± 132 0.99 kg/m^2^; hirsute N.12; with amenorrhea N.2, oligomenorrhoea N.13, regular cycles N.15) were diagnosed according to the Rotterdam ESHRE/ASRM 2003 criteria: presence of amenorrhea or oligomenorrhoea (<10 cycles/year), polycystic ovaries on ultrasonography, and hirsutism which was assessed according to Ferriman–Gallwey score (>8) (41). CTRL subjects (CA: 35.72 ± 0.55 yr; BMI: 24.08 ± 0.79 kg/m^2^) were women undergoing IVF because of tubal or unknown infertility causes, with normal endocrine exams and regular menstrual cycles. Exclusion criteria were the presence of tumors, endometriosis, coeliac disease, genetic or chronic diseases, Cushing syndrome, changes in thyroid function, hyperprolactinemia, and dysmorphisms. BMI was calculated as weight/height^2^ (kg/m^2^). All subjects were monitored by transvaginal ultrasonography (US) before oocyte retrieval according to the standard protocol in use at our Institution and the number of dominant follicles (diameter >17 mm) was recorded. All participants did not receive any additional treatment for at least 2 months before IVF, except for the ovarian stimulation therapy. Clinical details of PCOS and CTRL are reported in Table 1.

**Table 1:** Clinical features of subjects enrolled in the study.

	**CTRL (*N* = 36)**	**PCOS (*N* = 30)**
CA (years)	35.72 ± 0.55	34.43 ± 0.84
BMI (kg/m^2^)	24.08 ± 0.79	25.92 ± 0.99
Dominant Follicles (*N*)	3.92 ± 0.45	4.77 ± 0.48
E2 (pg/mL)	1275.86 ± 129.27	1894.10 ± 246.81^*^
Hirsute (*N*)	–	12
Amenorrhoea (*N*)	–	2
Oligomenorrhoea (*N*)	–	13
Regular cycles (*N*)	36	15

CA, chronological age; BMI, body mass index; E2, 17β-estradiol in serum. Data are mean ± SEM values.;

* *p < 0.05*.

### Hormonal Stimulation

Ovarian hormonal stimulation was conducted according to a long luteal gonadotropin-releasing hormone (GnRH) agonist depot protocol in order to obtain ovarian downregulation in all patients. After biochemical and instrumental confirmation of complete down-regulation (E2 concentrations <30 pg/ml and endometrial thickness ≤5 mm at transvaginal ultrasonography), recombinant FSH (rFSH) was administrated using a starting dose (first 5 days) tailored according to the patient's age and antral follicle count. From day 6 of ovarian stimulation, the dose of rFSH was adjusted according to ovarian response (monitored by transvaginal US and serum E2 concentrations). In case of appearance of leading follicles (of 17 mm and above), ovulation was triggered by injection of 10,000 IU human chorionic gonadotropin (hCG), 24 h after the last injection of rFSH, and 36 h later oocyte retrieval was performed by US-guided transvaginal aspiration.

### Collection of Follicular Fluid Samples and Granulosa Cell Isolation

FF was aspirated from follicles (14–22 mm in diameter) during the oocyte retrieval and was processed immediately after oocyte pickup. FF samples were centrifuged for 10 min at 1,500 × g at room temperature and then for 10 min at 3,000 × g at room temperature to completely remove red blood cells or detriments, then stored at −80°C for subsequent analyses. GC were isolated from the pellet obtained after the first centrifugation step which was resuspended in 10 mL of a 1:1 ratio mixture of Medium 199 (Sigma Aldrich, Inc., USA) and Hanks' balanced salt solution (Euroclone S.p.A., Italy). Then it was slowly layered on 10 mL of Lymphocyte separation medium (Ficoll-Paque Plus, GE Healthcare, UK) and centrifuged at 600 × g for 30 min at room temperature. The granulosa cells, collected at the interface, were drawn off using a sterile Pasteur pipette, then washed three times with Hanks' balanced salt solution and stored at −20°C until the RNA extraction.

### RNA Isolation and Reverse Transcription

Total RNA enriched in small RNAs was extracted from GC lysates and FF using the miRVana™ PARIS miRNA isolation Kit (Thermo Fisher Scientific, USA) according to the manufacturer's protocol. The RNA concentration of all samples was quantified by NanoDrop 1000 (Thermo Fisher Scientific, USA). The miRNAs from GC lysates and FF were reverse transcribed using the TaqMan® MicroRNA Reverse Transcription Kit (Thermo Fisher Scientific, USA) using a miRNA primer pool (see below) according to the manufacturer's protocol and the thermo-cycler T100 (Bio-Rad Laboratories, Inc., USA).

### RT-qPCR

Quantitative reverse transcription PCR (RT-qPCR) was performed in real-time using specific TaqMan® probes 20x (Thermo Fisher Scientific, USA) for hsa-miR146a (assay ID 000468), hsa-miR155 (assay ID 002623), hsa-miR320-3p (assay ID 002277), hsa-miR370-3p (assay ID 002275), hsa-miR486-5p (assay ID 478128_mir), U6 snRNA (assay ID 001973), and RNU48 (assay ID 001006) for the amplification of the endogenous controls. The small nuclear RNAs U6 and U48 were selected as reference genes. Experiments were performed in triplicate in optical 96-well reaction plates on the CFX96 Touch™ (Bio-Rad Laboratories, Inc. USA) thermo-cycler with TaqMan™ Universal Master Mix II, no UNG. Expression levels of the selected miRNA genes were normalized with respect to snU6 and snU48 expression levels in the same sample. Melting curves were analyzed to ensure that fluorescence signals reflected solely specific amplicons. The relative quantification analysis was determined using the 2^−ΔCt^ method (42).

### Biochemical Assays

HMGB1 concentrations in FF were assayed using a specific research ELISA kit (HMGB1 ELISA, Tecan Trading AG, Switzerland). The intra-assay coefficient of variation (CV) was 5.4%, and the inter-assay CV was 8.2%. The sensitivity of the method was <0.15 ng/mL. Insulin was measured in FF using a specific ultrasensitive ELISA kit (Mercodia Ultrasensitive Insulin ELISA, Mercodia AB, Sweden); the intra-assay CV was 6.5 and the inter-assay CV 7.1%. IL-6 was quantified in FF using an ultrasensitive ELISA method (Human IL6 Quantikine ELISA kit, R&D Systems, Inc. USA) according to the manufacturer's protocol; the intra-assay CV was 3.8% and the inter-assay CV 9.9%. The sensitivity of the method was <0.11 pg/mL. E2 concentrations, obtained from venous blood samples taken the same day as oocyte retrieval, were assayed with the ADVIA Centaur® Enhanced Estradiol Assay (Siemens AG 2010 2018, Germany), an automated, monoclonal, competitive, chemiluminescent immunoassay.

### miRNA-Target Gene Analysis

A list of experimentally validated direct target genes for each miRNA was obtained from an *in silico* analysis conducted on miRTarBase database (http://mirtarbase.mbc.nctu.edu.tw/php/index.php) in order to have confirmed that the miRNAs selected were tightly connected with the regulation of insulin sensitivity. Among the experimentally validated direct target genes of the analyzed miRNAs, those documented in the Literature to be related with insulin sensitivity have been reported in Table 2.

**Table 2:** miRNAs and their validated target genes related with insulin sensitivity obtained using miRTarBase.

**miRNA**	**miRBase ID**	**Validated target genes**
miR-146a	hsa-miR-146a-5p	*CFH, TLR2, FADD, TRAF6, IRAK1, BRCA2, BRCA1, FAF1, PA2G4, NFKB1, EGFR, FAS, ERBB4, SMAD4, TLR4, STAT1, ICAM1, SMAD2, PTGS2, CCL5, PTGES2, CXCL12, RAC1, COX2, SOS1, NOTCH2, SOX2, IL6, RHOA, LFNG, TGFB1, MIF, NOTCH1, CCND2, CCND1, RHO, RARB, NOS1*
miR-155	hsa-miR-155-5p	*MECP2, SOCS1, SHIP1, DET1, SMAD5, HIVEP2, JARID2, RHEB, PKN2, MYO10, RHOA, FOXO3, RUNX2, KBTBD2, KRAS, CYR61, SMAD2, SOX6, JUN, KDM3A, IL13RA1, BCL6, CARHSP1, MYBL1, NKX3-1, PRKAR1A, RAC1, ANXA2, CCND1, INPP5F, PAK2, UBQLN1, NFKB1, RAD51, MXI1, SOCS6, PTEN*
miR-320	hsa-miR-320a-3p	*IGF1R, HOXA10, VDAC1, MYC, ITGB3, RAC1, PDCD4, BMI1, ARF4, NRP1, NFAT, PTEN, RAB14, FOXM1, RUNX2, VEGFA, NOD2, FH, AR, HMGB1*
miR-370	hsa-miR-370-3p	*CPT1A, TGFBR2, FOXO1, LIN28A, SIK1, AQP3, CPT1B, FOXN3, HNRNPA1, IFNGR2, TRAF1, PYGO2, GPR146, PTEN, NUCKS1, WDTC1*
miR-486	hsa-miR-486-5p	*CD40, ARHGAP5, OLFM4, PIM1, IGF1R, CADM1, PCCA, FOXP1, SEC23IP, MACROD2, ARF6, FBN1, CDK4, SMAD2, FOXO1, PTEN, PIK3R1, HMGA1*

### Statistical Analysis

Standard statistical analysis was performed using the statistical package SPSS 24.0 for Windows (IBM Analytics, IBM Corporation 1994, 2017, USA) as appropriate. The Mann-Whitney U test for independent samples was used to study differences in miRNA levels between PCOS and CTRL. Pearson's linear regression or Spearman's rank were used for correlation analysis to investigate relationships among miRNAs, HMGB1, insulin, number of dominant follicles, E2, CA, BMI as appropriate. Only significant data are reported in the text. MiRNAs showing a *p* ≤ 0.05 were considered as differentially expressed. Data relative to miRNA are expressed as median and 25th-75th percentile, unless otherwise stated.

### Ethical Approval

The study was approved by the Ethical Committee of Reggio Emilia (Prot. No. PCOS2_15_17). All participants gave written informed consent in accordance with the Declaration of Helsinki.

## Results

### Analysis of miRNAs Levels in Granulosa Cells and Follicular Fluids

**Figure 1:**
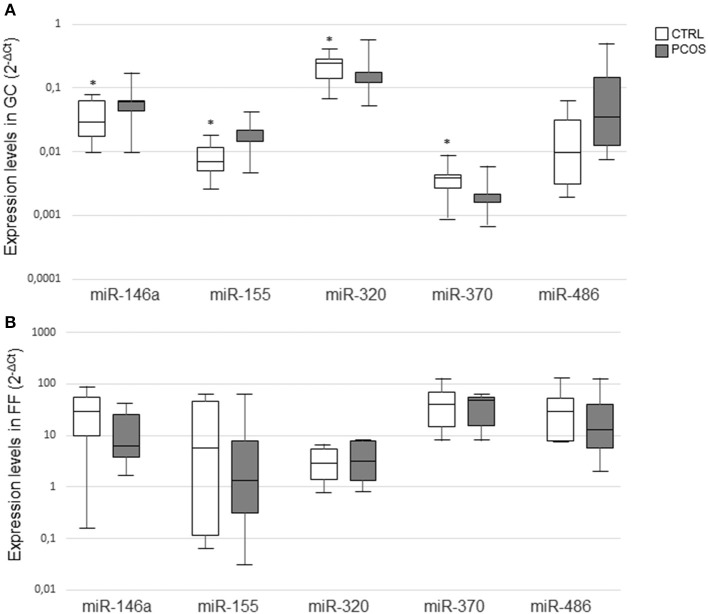
MiRNA expression levels in granulosa cells **(A)** and follicular fluids **(B)** from CTRL and PCOS. Results in box plots are shown as normalized expression (2^−ΔCt^) and the band inside the box is the median. The upper and lower whiskers represent the maximum and minimum values among data respectively. Data were compared using the Mann–Whitney *U*-test (*P ≤ 0.05).

Changes in miRNAs expression levels from GC of PCOS ovaries with respect to CTRL are reported in Figure 1A. MiR-146a and miR-155 were significantly upregulated, whereas miR-320 and miR-370 were downregulated in PCOS. MiR-486 did not differ significantly between the two groups but its expression level was increased in PCOS.

Changes in miRNAs from FF are represented in Figure 1B. In detail, miR-146a, miR-155, and miR-486 showed a trend to lower expression levels in PCOS, whereas miR-320 and miR-370 levels exhibited an opposite trend. However, none of these changes was statistically significant.

Overall, levels were lower in FF compared with those detected in GC lysates.

### Correlation Analyses in GC and in FF

#### Correlations Among miRNAs in GC

Within GC, significant correlations of miRNAs with other miRNAs are reported in Table 3.

**Table 3:** Significant correlations among miRNAs in granulosa cells in the entire group.

				**ρ**	***p*-value**
ENTIRE GROUP	miR-146a	vs.	miR-155	0.596	0.001
			miR-320	−0.285	0.029
	miR-155	vs.	miR-370	−0.432	0.001
			miR-486	0.515	0.034
	miR-320	vs.	miR-370	0.762	0.001

*ENTIRE GROUP, control subjects + Polycystic Ovary Syndrome patients; miR, microRNA; ρ, Spearman's correlation coefficient*.

In the entire group of subjects (PCOS and CTRL) miR-146a correlated positively with miR-155 (ρ = 0.596; *P* < 0.001) and negatively with miR-320 (ρ = −0.285; *P* = 0.029). MiR-155 correlated with miR-370 (ρ = −0.432; *P* = 0.001) and miR-486 (ρ = 0.515; *P* = 0.034). MiR-320 correlated miR-370 (ρ = 0.762; *P* < 0.001).

In the CTRL group, miR-320 correlated with miR-370 (ρ = 0.557; *P* = 0.002).

In PCOS, miR-146a correlated with miR-155 (ρ = 0.562; *P* = 0.001), and miR-320 with miR-370 (ρ = 0.491; *P* = 0.006).

#### Correlations of miRNAs in GC With Clinical Parameters and Biochemical Data

**Figure 2:**
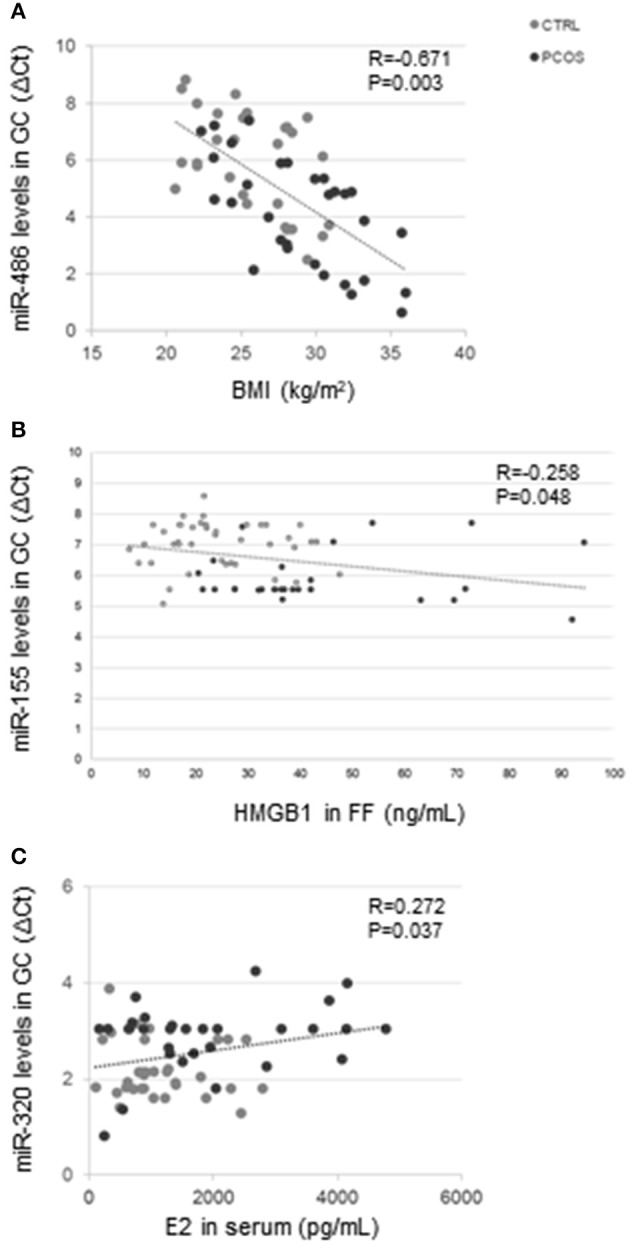
Correlation analysis in the entire group. MiR-486 levels in GC correlated with BMI (*R* = −0.671; *P* = 0.003) **(A)**. MiR-155 levels in GC correlated with HMGB1 concentrations (*R* = −0.258; *P* = 0.048) **(B)**, and miR-320 levels in GC correlated with serum E2 concentrations (*R* = 0.272; *P* = 0.037) **(C)**.

In the entire group of subjects (PCOS and CTRL) miR-486 correlated with BMI (*R* = −0.671; *P* = 0.003) (Figure 2A), and both miR-155 (*R* = −0.259; *P* = 0.047) and miR-370 (*R* = 0.260; *P* = 0.047) correlated with the number of dominant follicles. MiR-155 correlated negatively with HMGB1 (*R* = −0.258; *P* = 0.048) in FF (Figure 2B). MiR-155 (*R* = 0.411; *P* = 0.001), miR-320 (*R* = −0.406; *P* = 0.001), and miR-370 (*R* = −0.510; *P* < 0.001) correlated all with insulin concentrations. MiR-320 correlated with serum E2 concentrations (*R* = 0.272; *P* = 0.037) (Figure 2C).

In PCOS, miR-486 correlated both with chronological age (*R* = −0.676; *P* = 0.032), BMI (*R* = −0.644; *P* = 0.045), and serum E2 concentrations (*R* = 0.741; *P* = 0.014). MiR-146a correlated with HMGB1 (*R* = 0.333; *P* = 0.072) in FF.

#### Correlations Among miRNAs in FF

In the entire group of subjects miR-155 positively correlated with miR-370 (*R* = 0.611; *P* = 0.027). MiR-320 correlated both with miR-370 (*R* = 0.727; *P* = 0.005) and miR-486 (*R* = 0.827; *P* = 0.001); miR-370 correlated with miR-486 (*R* = 0.781; *P* = 0.002).

In the CTRL group miR-370 correlated both with miR-155 (R = 0.910; P = 0.032), and miR-486 (*R* = 0.985; *P* = 0.002).

In PCOS, miR-320 correlated both with miR-370 (*R* = 0.800; *P* = 0.017) and miR-486 (*R* = 0.956; *P* = 0.001). MiR-486 and miR-370 were also related (*R* = 0.740; *P* = 0.036).

#### Correlations of miRNAs With Clinical Parameters in FF

In the entire group, miR-155 correlated with BMI (*R* = 0.366; *P* = 0.039).

In the CTRL group, miR-155 correlated both with CA (*R* = 0.512; *P* = 0.030) and BMI (*R* = 0.492; *P* = 0.038).

In PCOS, miR-486 correlated with E2 (*R* = −0.661; *P* = 0.038), miR-146a with insulin concentrations (*R* = 0.548; *P* = 0.042), and miR-155 correlated with IL6 (*R* = −0.587; *P* = 0.027).

## Discussion

This study showed an upregulation of miR-146a, miR-155, miR-486 levels and a downregulation of miR-320 and miR-370 levels in GC from PCOS patients compared with a CTRL population. Although no significant changes were found in FF, miR-146a, miR-155, and miR-486 showed lower levels in PCOS, whereas miR-320 and miR-370 levels exhibited an opposite trend. These miRNAs showed relationships with BMI, serum E2 concentrations, number of dominant follicles, insulin, and HMGB1 concentrations in FF.

We are aware that this study has some limitations. In particular, the fact that all analyses were performed after ovarian hormonal stimulation that might induce changes; however, both PCOS and CTRL subjects underwent the same stimulation protocol, thus differences between the two groups must be considered as such. Furthermore, for ethical reasons, GC and FF can be easy to obtain as a secondary product during IVF procedures.

The finding of increased miR-146a in GC from PCOS is consistent with reports under conditions of insulin resistance in pancreatic β-cells after exposure to saturated fatty acids (43) and in serum from type 2 diabetic patients (44). In the miRTarBase database it is reported that about 54% of the experimentally validated direct target genes for miR-146a are related with insulin sensitivity (Table 2). A downregulation of miR-146a has been associated with increased protein-tyrosine phosphatase non-receptor-type 1 (PTPN1), an inhibitor of the insulin receptor (45). As we previously described reduced insulin content in FF in PCOS (17), it could be speculated that insulin uptake might be enhanced in GC in PCOS women, due to enhanced insulin sensitivity possibly associated with reduced PTPN1. Interestingly, miR-146a upregulation was reported in patients with premature ovarian failure, and associated with enhanced ovarian GC apoptosis (46). This could be further supported by our finding of a positive correlation between miR-146a and HMGB1, as HMGB1 has been described to be released during apoptosis (47). Furthermore, we previously described relationships of HMGB1 with glucose metabolism and inflammation both in cystic fibrosis and PCOS (17, 18). Interestingly, miR-146a was found to be positively correlated also with miR-155. Both these miRNAs are recognized as possible regulators of the *FOXO1* gene, a key downstream mediator of the insulin signaling cascade (25).

MiR-155 is considered a multifunctional miRNA and is strongly related with inflammation, insulin resistance, obesity, and steroidogenesis (33, 48, 49) reflecting the main features of PCOS. Furthermore, following *in-silico* analysis using miRTarBase, about 21% of experimentally validated direct target genes of miR-155 are related with insulin sensitivity (see Table 2). In this study miR-155 was found upregulated in PCOS and correlated with both HMGB1 and insulin concentrations in FF, supporting a possible involvement of miR-155 in the regulation of inflammation and ovarian insulin sensitivity. As mentioned above, miR-155 is predicted to target the *FOXO1* gene (25), which also controls genes involved in gluconeogenesis and adipogenesis, metabolic processes related with insulin sensitivity. The correlation with BMI, CA and IL-6 is in agreement with inflammation increasing with BMI and age (50, 51). The correlations with other miRNAs confirm the existence of a network of miRNAs regulating a network of corresponding genes involving in the regulation of insulin sensitivity within the ovary.

MiR-320 and miR-370 were decreased in GC from PCOS and correlated with insulin concentrations. In serum these miRNAs are known to regulate insulin sensitivity (38, 52). Within miRTarBase database, ~14–43% of validated target genes for these miRNAs are found among genes known to regulate insulin sensitivity (see Table 2). Our data suggested also that they play a role in regulating insulin sensitivity within the ovary. The relationships of miR-320 with E2 and of miR-370 with the number of dominant follicles suggests a role in ovarian function. Interestingly, miR-320 and miR-370 have been described to regulate mediators downstream the follicle-stimulating hormone (FSH) receptor that plays a key role in folliculogenesis, and oocyte maturation (53). Some of these mediators are shared by the insulin receptor signaling pathway, therefore, a cross talk between the two receptors could be hypothesized.

MiR-320 is currently considered as a promising target for the treatment of type-2 diabetes mellitus (34) and regulates the expression levels of the p85 subunit of the PI3K which enhances adipocyte insulin sensitivity in obesity (52). Furthermore, miR-320 has been described to downregulate the expression of *IGF-1* and its receptor (52, 54). MiR-370 has been shown to be a *FOXO1* gene regulator in prostatic cancer cells (55, 56). It is also described as a regulator of Insulin receptor substrate 1 (IRS1), an insulin-signaling scaffold-protein (37, 38, 55).

MiR-486 has been previously described as a *FOXO1* gene regulator and thus is related with insulin sensitivity (34, 57, 58). Furthermore, following *in-silico* analysis using miRTarBase, about 27% of validated target genes of miR-486 are related with insulin sensitivity (see Table 2). Changes were not significant in PCOS, although it was increased with respect to controls both in GC and in FF levels. MiR-486 was correlated with BMI, chronological age, and serum E2 concentration in PCOS, thus suggesting that aging and adiposity were related with ovarian function.

In conclusion, this study highlighted that some miRNAs are of importance in PCOS within the ovary, and show relationships with insulin levels, ovarian insulin sensitivity and inflammation. GC and FF, due to their close proximity with the oocyte and its nurse cells reflect the secretory and metabolic activities of oocytes and follicle niche. Overall this study offers new knowledge on PCOS within the ovarian microenvironment, providing a new insight into insulin sensitivity even if insulin *per se* and insulin sensitivity need to be further investigated and understood within the ovary.

## Data Availability Statement

The datasets generated for this study are available on request to the corresponding author.

## Ethics Statement

The studies involving human participants were reviewed and approved by the Local Ethical Committee of Reggio Emilia (Prot. No. PCOS2_15_17). Affiliation: AUSL-IRCCS di Reggio Emilia, Viale Umberto I n° 50, 42123, Reggio Emilia, Italy. The patients/participants provided their written informed consent to participate in this study.

## Author Contributions

MS: conceptualization, supervision, and funding acquisition. MS, FC, CC, and GL: methodology. FC and CC: formal analysis and writing—original draft preparation. PL and CS: investigation. SA, MS, and AN: resources. PL, CS, FC, CC, and AN: data curation. MS and SA: visualization. FC, CC, MS, and AN: project administration.

### Conflict of Interest

The authors declare that the research was conducted in the absence of any commercial or financial relationships that could be construed as a potential conflict of interest.
